# The role of nutrition, intimate partner violence and social support in prenatal depressive symptoms in rural Ethiopia: community based birth cohort study

**DOI:** 10.1186/s12884-018-2009-5

**Published:** 2018-09-15

**Authors:** Yitbarek Kidane Woldetensay, Tefera Belachew, Hans Konrad Biesalski, Shibani Ghosh, Maria Elena Lacruz, Veronika Scherbaum, Eva Johanna Kantelhardt

**Affiliations:** 1 Institute of Biological Chemistry and Nutrition (140a), University of Hohenheim, Stuttgart, Germany; 2 Food Security Center, University of Hohenheim, Stuttgart, Germany; 3 Department of Population and Family Health, College of Health Sciences, Jimma University, Jimma, Ethiopia; 4 Tufts University, Freidman School of Nutrition Science and Policy, Boston, USA; 5 Department of Gynecology, Faculty of Medicine, Martin-Luther University, Halle, Germany; 6 Institute of Medical Epidemiology, Biostatistics, and Informatics, Faculty of Medicine, Martin-Luther University, Halle, Germany

**Keywords:** Prenatal depression, Household food insecurity, Anaemia, Intimate partner violence, Social support, PHQ-9, Ethiopia

## Abstract

**Background:**

Depression during pregnancy has far-reaching adverse consequences on mothers, children and the whole family. The magnitude and determinants of prenatal depressive symptoms in low-resource countries are not well established. This study aims to describe the prevalence of prenatal depressive symptoms and whether it is associated with maternal nutrition, intimate partner violence and social support among pregnant women in rural Ethiopia.

**Methods:**

This study is based on the baseline data from a large prospective, community-based, birth cohort study conducted in the South Western part of Ethiopia from March 2014 to March 2016. A total of 4680 pregnant women were recruited between 12 and 32 weeks of gestation. Depressed mood was assessed using the Patient Health Questionnaire (PHQ-9) scale and a cut off of ≥8 was taken to define prenatal depressive symptoms. Data collection was conducted electronically on handheld tablets and submitted to a secured server via an internet connection. Bivariate and multivariate logistic regression analyses were computed using IBM SPSS version 20 software.

**Result:**

The community based prevalence of depressive symptoms during pregnancy was 10.8% (95%Confidence Interval (CI): 9.92–11.70). Adjusting for confounding variables, moderate household food insecurity (OR 1.74; 95% CI: 1.31–2.32), severe household food insecurity (OR 7.90; 95% CI: 5.87–10.62), anaemia (OR = 1.30; 95% CI: 1.04–1.61) and intimate partner violence (OR 3.08; 95% CI: 2.23–4.25) were significantly associated with prenatal depressive symptoms. On the other hand, good social support from friends, families and husband reduced the risk of prenatal depressive symptoms by 39% (OR 0.61; 95% CI: 0.50–0.76).

**Conclusion:**

Prenatal depressive symptomatology is rather common during pregnancy in rural Ethiopia. In this community based study, household food insecurity, anaemia and intimate partner violence were significantly associated with prenatal depressive symptoms. Good maternal social support from friends, families and spouse was rather protective. The study highlights the need for targeted screening for depression and intimate partner violence during pregnancy. Policies aimed at reducing household food insecurity, maternal anaemia and intimate partner violence during pregnancy may possibly reduce depression.

## Background

Major depression is the leading cause of the global burden of disease today [1]. It is also the most prevalent psychiatric disorder during pregnancy [2]. Prenatal depression can lead to serious health risks for both the mother and infant [3, 4]. A recent systematic review revealed that in low- and lower-middle-income countries (LMICs) average prevalence of perinatal mental disorder (25.3%: 95% CI: 21.4–29.6%) was considerably higher than the 7–15% prevalence in high-income countries [2]. Nonetheless, low-income countries assign about 0.5% of their health budget to mental health while high-income countries devote 5.1%, an amount, still disproportionately small (given the prevalence and impact of mental disorders), to implement a series of highly cost-effective interventions [1].

In Ethiopia the prevalence of prenatal depression varies widely based on the instruments used and study settings. A 12% prevalence of prenatal depression was reported using PHQ-9 scale [4], 23% using Beck Depression Inventory (BDI) scale [5], 25% using Edinburgh Postnatal Depression Scale [6] and 31.5% using WHO Self-Reported Questionnaire with 20 items (SRQ-20) [7]. A community based study showed a 12% prevalence of common mental disorder during pregnancy [4]. Whereas, health facility based studies revealed 23–31.5% prevalence [5–7].

Based on previous research findings in low, middle and high income countries, socio-demographic factors such as age [5, 8], income [9] and educational attainment [7] were identified as factors affecting prenatal depressive symptoms. Clinical factors includes previous depression [8], concomitant high anxiety in pregnancy (Strewart et al., 2003) and a history of miscarriage and induced abortion [10, 11]. Studies also showed that household food insecurity [12] and anaemia [13] are identified as nutrition related factors for prenatal depression. A number of studies also indicated that intimate partner violence is another factor associated with depression during pregnancy [14, 15].

Several studies have shown a role of nutrition in mental distress, and they mostly documented the psychological effects of nutrient deficiencies. These studies indicated that deficiencies in folate, vitamin B12, calcium, iron, selenium, zinc, and polyunsaturated fatty acids (PUFAs) are associated with depression. Particularly, omega-3 fatty acids are getting special attention regarding their efficacy in depression treatment [16]. Nutrition is a modifiable risk factor, and therefore is possible to be improved with targeted programs in addition to support programs to reduce maternal distress [17].

Studies exploring the association between maternal nutrition and prenatal depression are still inconclusive and the limited studies available did not control for important variables such as intimate partner violence and social support [18]. In Ethiopia, though the prevalence and determinants of intimate partner violence is well studied in the general population, there are limited data describing its association with prenatal depression [19]. This study aims to describe the prevalence of prenatal depression and whether it is associated with maternal nutrition, intimate partner violence and social support among pregnant women in rural Ethiopia.

## Methods

This study utilizes baseline data from a prospective, community-based, quasi-experimental birth cohort study within Empowering New Generations to Improve Nutrition and Economic opportunities (ENGINE) program. ENGINE is a USAID funded program, which aims to improve nutritional status of mothers’ and young children in Ethiopia through a multi-sectoral approach targeting health, nutrition and agriculture. The ENGINE birth cohort study was led by Tufts University and aimed to investigate the benefits of an integrated nutrition program and its co-location with agricultural growth program on household agricultural production and productivity, food security, diet diversity, socio-economic status and livelihoods, as well as health status, anthropometry and hemoglobin for mother and her child.

This study had an open cohort design, with rolling recruitment and follow up of pregnant women for a period of two years. The study was conducted from March 2014 to March 2016 in three Districts (Woliso, Tiro-Afeta and Gomma) in the South Western part of Ethiopia. A total of 4680 pregnant women were recruited between 12 to 32 weeks of gestation and followed up until 12 months postpartum. Data was collected once during pregnancy for all women (twice for those in the first trimester at recruitment), at birth, and then every three months until the child was 12 months old. Data collection was conducted by trained nurses electronically using Open Data Kit (ODK) software on handheld tablets and submitted to a secured server via an internet connection. Ethical clearance was obtained from Jimma University ethical review board. Informed written consent was obtained from all individual participants included in the study. All interviews were conducted in private and confidentiality was ensured for each study participants.

## Measurements

### Depressive symptoms

Prenatal depressive symptoms were assessed using the patient health questionnaire (PHQ-9). The PHQ-9 is a 9-item self-administered questionnaire designed to evaluate the presence of depressive symptoms during the prior two weeks [20]. Each of the nine items can be scored from 0 (not at all) to 3 (nearly every day). Thus, total score can range from 0 (absence of depressive symptoms) to 27 (most severe depressive symptoms). This instrument had been validated for Afaan Oromo Language in a similar population prior to the commencement of the ENGINE birth cohort study and possessed good psychometric properties. A PHQ-9 score of 8 or above was taken as a cut off to define prenatal symptomatology [21].

### Nutritional status

Mid upper arm circumference (MUAC) was used to estimate maternal nutritional status. It was measured three times at each visit at the midpoint between the tip of the shoulder and the elbow of the left upper arm using inelastic adult MUAC tape. The average of three MUAC measurements was calculated and then categorized as normal or low MUAC. A MUAC of less than 23 cm was considered to be a sign of poor nutrition [22].

### Anaemia

Haemoglobin concentration was measured using HemoCue® Hb 301 system for mobile screening. One drop of blood was collected in a HemoCue microcuvette and the haemoglobin concentration was read directly in the field. Anaemia was defined as haemoglobin concentration < 11 g/dl after adjusted for altitude and pregnancy to get the sea level value according to the method described by Cohen and Hass [23].

### Household food insecurity

The household food insecurity was measured using the Household Food Insecurity Access Scale [24]. The index women were asked nine questions (yes/no) to determine if anyone in their household had experienced problems of food access over four weeks preceding the interview. An affirmative response to any of the nine questions was followed by a question to determine how often the condition happened: rarely (1–2 times), sometimes (3–10 times), and often (> 10 times). Responses were coded as 0 = never (i.e., no experience), 1 = rarely, 2 = sometimes, or 3 = often. Household food insecurity was categorized into four severity levels: food secure, mildly food insecure, moderately food insecure, and severely food insecure as per the algorithm described by Coates et al. [24].

### Socio-demographic factors

Educational status of the index mother was dichotomized as above primary school and primary or below for analysis purpose. Marital status was dichotomized into married (married monogamous and married polygamous) and unmarried (single, widowed, divorced, and separated). Religion was categorized into three as Protestant and Catholic, Muslim and Orthodox (only 3 respondents were follower of traditional religion or pagan and hence were not separately analyzed).

### Obstetric related risk factors

Gravidity, gestational age, acute illnesses in the past two weeks, place of previous delivery and history of previous antenatal care visits; previous child death and spontaneous abortion were considered obstetric-related risk factors. Gravidity was categorized into primi-gravida (first pregnancy), multi-gravida (2–4 pregnancy experience) and grand-multi-gravida (five or more than five pregnancy experiences). While gestational age was categorized into three as first trimester (up to 12 weeks of gestation), second trimester (13–26 weeks of gestation) and third trimester (above 26 weeks of gestation).

### Intimate partner violence (IPV)

A screening tool called HITS (Hurt, Insult, Threaten and Scream) was applied to assess intimate partner violence. This screening tool measures the emotional (psychological) aspects of intimate partner violence. The scale has four items and each item was scored on a scale of 1 (never) to 5 (frequently) and later the sum score was computed. A total score of > 10 is suggestive of IPV [25].

### Maternal social support

Maternal Social support was measured using the Maternity Social Support Scale (MSSS) developed by Webster and colleagues [26]. The scale contains six items. Each item has measured on a five-point Likert scale and a total score of 30 was possible. We classified social support into two categories based on the mean score; below mean and mean or above mean score.

## Data analysis

The data was analyzed using SPSS version 20. Bivariate and multivariate logistic regression analyses were computed to examine the relationship between the independent variables and prenatal depressive symptoms. The binary form of the dependent variable was coded as “1” for prenatal depressive symptoms (PHQ-9 score > = 8) and “0” for the absence (PHQ-9 score < 8). First binary logistic regression analyses were conducted between each individual independent variable and prenatal depressive symptoms. The findings were reported using unadjusted Odds Ratios (OR) and its 95% confidence interval (CI).

Then a full model including the nutritional (household food insecurity, anaemia, MUAC, fasting, nutrition related knowledge) socio-demographic (age, religion, marital status, family size and wealth index) and other confounders (obstetric factors, acute illnesses, social support, chat chewing practices and intimate partner violence) were fitted using a multivariate binary logistics regression to identify the independent predictors of prenatal depressive symptoms. Adjusted odds ratios (OR) and their 95% CI were presented as indicators of strength of association. A *p*-value of 0.05 or less was used to determine the cut-off points for statistical significance.

## Results

### Characteristics of study participants

All recruited 4680 pregnant women between March 2014 and March 2016 were included in the final analysis. The median age of study participants was 26 years [inter-quartile range (IQR) 22, 30]. More than half of the pregnant women (55.2%) were illiterate and only 241(5.1%) of the respondents had completed secondary education or higher. Just over two-third (67.3%) of the respondents were Muslim and 97.7% were married. Participants’ characteristics are presented in Table 1.

**Table 1:** Characteristics of the study participants, Ethiopia, 2016

Variables		Number	Percent
Age	Less than 25 years	1615	34.5
	25–35 years	2831	60.5
	Above 35 years	234	5.0
	Median (IQR)	26 (22–30)	
Religion	Muslim	3148	67.3
	Orthodox	1057	22.6
	Protestant & Catholic	472	10.1
	Missing	3	0.1
Marital status	Married	4572	97.7
	Unmarried	108	2.3
Education	Illiterate	2585	55.2
	Primary school	1491	31.9
	Junior secondary school	363	7.8
	Secondary and above	241	5.1
	Median (IQR)	0 (0–4)	
Family size	Less than five	2216	47.4
	Five or more	2464	52.6
	Median (IQR)	5 (3–6)	
Wealth quintile	Lowest	928	19.8
	Second	957	20.4
	Middle	863	18.4
	Fourth	986	21.1
	Highest	934	20.0
Household Food Insecurity	Secure	1600	34.2)
	Mildly insecure	600	12.8
	Moderately insecure	1846	39.4
	Severely insecure	634	13.5
Fasting	Yes	2428	51.9
	No	2252	48.1
Anaemia	Greater or equal to 11 g/dl	3409	72.84
	Less than 11 g/dl	1271	27.2
Mid-upper Arm Circumference (MUAC)	Greater or equal to 23 cm	2771	59.2
	Less than 23 cm	1909	40.8
	Median (IQR)	23.30 (22.07–24.57)	
Chat chewing	Yes	690	14.7
	No	3990	85.3
Nutrition related knowledge	Yes	315	6.7
	No	4365	93.3
Antenatal care (previous pregnancy)	No ANC	1626	34.7
	One to three times	1924	41.1
	Greater than four visits	1859	39.7
Gravidity	Primi-gravida	608	13.0
	Multigravida	1924	41.1
	Grand multigravida	2148	45.9
Gestational age	First trimester	164	3.5
	Second trimester	2869	61.3
	Third trimester	1647	35.2
History of child death	Yes	1214	25.9
	No	3466	74.1
Previous spontaneous abortion	Yes	539	11.5
	No	4141	88.5
Acute illness	Yes	1203	25.7
	No	3477	74.3
Social participation	Yes	2886	61.7
	No	1794	38.3
	Median (IQR)	1.0 (0–1.0)	
Maternal social support	Good support	2485	53.1
	Poor support	2195	46.9
Intimate partner violence	Yes	232	5.0
	No	4448	95.0

### Prevalence of prenatal depressive symptoms

A total of 506 pregnant women had a PHQ-9 score ≥ 8, yielding a crude depressive symptom prevalence rate of 10.81% (95% CI: 9.92–11.70). The prevalence of depressed mood in pregnant women is depicted in Table 2. The prevalence was higher among pregnant women age above 35 years (11.8% versus 8.6% for younger women), unmarried (26.9% versus 10.4% for married) and illiterate (11.4% versus 5.0% for secondary school and above). Nearly 13 % of Muslim pregnant women were in depressed mood compared to 6.7% for Orthodox and 6.1% for Protestants and Catholic Christians. The prevalence of prenatal depressive symptoms increased with household food insecurity severity; 34.4% of mothers in severely food insecure households were suffering from depressed mood compared to 4.8% in food secure households (*p* < 0.001). Moreover, the prevalence was higher among anaemic (14.2% versus 9.5% for without anaemia) and under-nourished (12.4% versus 9.7% for well-nourished, *p* = 0.005) pregnant women. The depressive symptoms prevalence increased with gestational age which is 8.7%, 10.2% and 12.0% (*p* = 0.039) for first, second and third trimester respectively. The severity of the depressed mood was also increased with gestational age with mean values of 2.98 (+ 3.05), 3.03 (+ 3.50) and 3.26 (+ 3.61) for the first, second and third trimester respectively. Prenatal depressive symptomatology was more prevalent among mothers who encountered intimate partner violence (29.7% versus 9.8% for mothers with no IPV experience).

**Table 2:** Variables associated with prenatal depressive symptoms, Ethiopia, 2016

Variables		Depressive Symptoms Number (%)	Unadjusted OR (95%CI)	*p-value*	Adjusted OR (95%CI)	*p-value*
Age	Less than 25 years	139 (8.6)	**0.59 (0.39–0.90)**	**0.01**	0.77 (0.47–1.26)	0.294
	25–35 years	335 (11.8)	0.85 (0.57–1.25)	0.41	0.85 (0.55–1.32)	0.474
	Above 35 years	32 (13.7)	1.0		1.0	
Religion	Orthodox	71 (6.7)	0.91 (0.58–1.42)	0.68	0.80 (0.54–1.19)	0.272
	Protestant & Catholic	29 (6.1)	**2.05 (1.58–2.67)**	**< 0.001**	0.85 (0.50–1.45)	0.557
	Muslim	405 (12.9)	1.0		1.0	
Marital status	Married	477 (10.4)	1.0		1.0	
	Unmarried	29 (26.9)	**3.15 (2.04–4.87)**	**< 0.001**	**2.65 (1.59–4.44)**	**< 0.001**
Education	Primary or below	465 (11.4)	**1.77 (1.27–2.46)**	**< 0.001**	1.07 (0.74–1.56)	0.707
	Above primary	41 (6.8)	1.0		1.0	
Family size	Less than five	198 (8.9)	1.0		1.0	
	Five or more	308 (12.5)	**1.46 (1.21–1.76)**	**< 0.001**	**1.36 (1.08–1.71)**	**0.010**
Wealth index	Lowest	90 (9.7)	0.81 (0.60–1.08)	0.148		
	Second	125 (13.1)	1.13 (0.86–1.48)	0.397		
	Middle	78 (9.0)	0.74 (0.55–1.01)	0.059		
	Fourth	103 (10.4)	0.87 (0.66–1.16)	0.354		
	Highest	110 (11.8)	1.0			
Address	Gomma	209 (13.4)	**2.26 (1.76–2.90)**	**< 0.001**	**3.04 (2.04–4.53)**	**< 0.001**
	Tiro-Afeta	197 (12.6)	**2.11 (1.64–2.71)**	**< 0.001**	**2.02 (1.34–3.05)**	**0.001**
	Woliso	100 (6.4)	1.0		1.0	
Household Food Insecurity	Secure	76 (4.8)	1.0		1.0	
	Mildly insecure	29 (4.8)	1.02 (0.66–1.56)	0.309	0.84 (0.54–1.31)	0.445
	Moderately insecure	183 (9.9)	**2.21 (1.67–2.91)**	**0.001**	**1.74 (1.31–2.32)**	**< 0.001**
	Severely insecure	218 (34.4)	**10.51(7.92–13.94)**	**< 0.001**	**7.90 (5.87–10.62)**	**< 0.001**
Fasting	Yes	256 (10.5)	1.0			
	No	250 (11.1)	1.06 (0.88–1.27)	0.539		
Haemoglobin Concentration	11 g/dl or more	325 (9.5)	1.0		1.0	
	Less than 11 g/dl	181 (14.2)	**1.58 (1.30–1.91)**	**< 0.001**	**1.30 (1.04–1.61)**	**0.019**
Mid-upper Arm Circumference (MUAC)	Greater or equal to 23 cm	270 (9.7)	1.0		1.0	
	Less than 23 cm	236 (12.4)	**1.31 (1.09–1.57)**	**0.005**	0.96 (0.78–1.18)	0.692
Chat chewing	Yes	104 (15.1)	**1.58 (1.26–2.00)**	**< 0.001**	0.94 (0.72–1.23)	0.638
	No	402 (10.1)	1.0		1.0	
Nutrition Related knowledge	Yes	43 (13.7)	1.0			
	No	463 (10.6)	0.75 (0.54–1.05)	0.094		
Maternal social support	Good support	185 (7.4)	1.0		1.0	**< 0.001**
	Poor support	321 (14.6)	**2.13 (1.76–2.58)**	**< 0.001**	**1.63 (1.31–2.02)**	
Intimate partner violence	Yes	69 (29.7)	**3.38 (2.59–4.42)**	**< 0.001**	**3.08 (2.23–4.25)**	**< 0.001**
	No	437 (9.8)	1.0		1.0	

### Socio-demographic factors

Prenatal depressive symptoms was significantly associated with marital status (*p* < 0.001). Unmarried pregnant women were 2.65 times more likely to develop depressive symptoms than their married counterparts (AOR = 2.65; 95%CI: 1.59–4.44). Pregnant women in households with more than five family size are 1.36 times (AOR = 1.36; 95%CI: 1.08–1.71) more at risk of depressive symptoms than those living in small family size households. Similarly, geographic location was important with women living in some districts being more likely to exhibit depressive symptoms. Pregnant women in Gomma and Tiro-Afeta districts faced 3.04 times (AOR = 3.04; 95%CI: 2.04–4.53) and 2.02 times (AOR = 2.02; 95%CI: 1.34–3.05) higher risk of depressive symptoms than those living in Woliso district. None of the remaining socio-demographic variables shown in Table 2 were associated with an increased prevalence of major depressive symptoms (Table 2).

### Nutrition related factors

After adjusting for confounding variables, women with moderate and severe household food insecurity had 1.74 (AOR = 1.74; 95% CI: 1.31–2.32) and 7.90 (AOR 7.90; 95% CI: 5.87–10.62) times higher risk of prenatal depressive symptoms respectively than women who were living in food secure households. Similarly, anaemic pregnant women were at higher risk of prenatal depression than those with normal haemoglobin concentration (AOR = 1.30; 95% CI: 1.04–1.61). Examining the crude odds ratios, we found that prenatal depressive symptoms was positively associated with both undernutrition assessed by low MUAC (AOR = 1.31; 95%CI: 1.09–1.57) and chat chewing (AOR = 1.58; 95%CI: 1.26–2.00). However, this relationship disappeared when adjusted for all other variables in the final model (Table 2).

### Intimate partner violence and maternal social support

As shown in Table 2, depressive symptomatology was more likely among participants who encountered higher intimate partner violence (AOR = 3.08; 95%CI: 2.23–4.25) and poor social support from spouse, families and friends (AOR = 1.63; 95%CI: 1.31–2.02).

## Discussion

The key contribution of this paper is to show the prevalence of prenatal depressive symptoms and its association with nutrition related factors, intimate partner violence and social support in rural Ethiopia. This finding has important implications, particularly in Ethiopia, where the burden of mental health diseases and intimate partner violence are high, resource allocation towards mental health care is poor with four psychiatrists per 10,000,000 population [27], inadequate nutritional status in pregnancy is still a considerable public health burden and both nutrition and intimate partner violence are modifiable risk factors.

The relationship between IPV, depression and food insecurity are all bidirectional and social support plays a buffering and protective role in this link. Depression is the most common mental health consequences of IPV [28, 29]. Previous studies indicated that women who experience IPV have about four times greater risk of depression than women who do not experience IPV. On the other hand, depression is associated with the use of hostility, insult, and threat in marital interactions [30, 31]. When we see the pathway between IPV and household food insecurity, previous research demonstrated that it is mediated by depression [32].

Poverty is one of the key contributors to intimate partner violence [33]. Since poverty is inherently stressful, it has been argued that intimate partner violence may result from stress and that poorer men have fewer resources to reduce stress. Poverty as it impairs purchasing power, results in household food insecurity. IPV may affect the couple’s capacity to organize the home environment and manage the resources available in order to guarantee the food and nutrition security of the family. Looking this link from household food insecurity side, a broader anthropological conceptualization of food insecurity posits that acute or chronic exposure to periods of food uncertainty can influence mental and physical health outcomes. Social support plays a buffering role for both depressive symptoms and IPV. Social support from family or friends buffers the effects of environmental stressors such as IPV and poverty and could decrease individual’s vulnerability to depression [34].

Consistent with previous studies in low, middle and high income countries, this study revealed that household food insecurity is strong predictor of prenatal depressive symptoms [12, 35–38]. Food insecurity by itself is a stressful life event, and the occurrence of stressful events was shown to affect the hypothalamic-pituitary-adrenocortical (HPA) axis. It is also known that hypothalamic dysfunction was linked to the onset and recurrence of depression [39].

Moreover, previous studies indicated that food insecurity was linked to specific nutrient deficiencies, which were also associated with depressive symptoms [16, 40]. These studies showed that food insecurity influences prenatal depression through deficiencies in energy, vitamin B12, Selenium or folic acid. Yet, another study also indicated that low-income women with depressive symptoms and life stressors represent an at-risk group for low diet quality during pregnancy and hence the link between depression and nutrient deficiencies is bidirectional [41]. Using nationally representative data and a number of different modeling approaches, Noonan and colleagues found robust evidence that maternal depression has adverse effects on household food insecurity [42]. Hence, the association between depression and food insecurity is also bidirectional.

In this study, pregnant women with depressive symptoms had lower haemoglobin levels than women without depressive symptoms. In agreement with our findings, previous observational studies generally established that anaemia is associated with depression [13, 43]. However, a placebo and high-iron diet controlled supplementation trial among female participants in high income countries found no association between depression and anaemia [44].

The relationship and direction of the relationship between depression and maternal anaemia remains unclear and still needs further investigation. However, there are different hypotheses about the mechanisms linking anaemia with depression. Iron is a co-factor in synthesis of tyrosine and tryptophan. Tyrosine and tryptophan are precursors for the neurotransmitters dopamine, norepinephrine and serotonin [45]. The traditional monoamine hypothesis of depression speculates that low dopamine, norepinephrine, and serotonin concentrations may result in depression [46]. In addition, iron is a cofactor for the reaction leading to the production and secretion of glutamate [47]. The glutamate hypothesis of depression has posited that dysregulation of the glutamatergic system results in depression [48].

In congruence with other previous studies, we found a statistically significant association between intimate partner violence and prenatal depressive symptoms [14, 15, 49]. Because of fear of stigmatization, battered women often experience feelings of shame, isolation and entrapment and did not communicate openly to others that violence occurred to them by their spouses [50]. This results in lack of support from friends and families and rather leads to more depression.

Respondents with prenatal depressive symptoms reported poorer maternal social support compared to their counterparts. Our finding is consistent with the suggestion that social support may safeguard the adverse effects of prenatal psychological distress on birth outcomes [51, 52]. The buffering hypothesis of social support postulated that the potential pathogenic effect of stressful events is reduced when support is accessible [53].

The prevalence of depressive symptoms during pregnancy in our study was lower compared to previous prevalence reports in Ethiopia [4–7, 54]. The relatively lower prevalence in our study probably reflects the fact that this is a population based study while the prior studies were health facility based. It could be postulated that the difference in rates could be due to different population sub-groups, for example in the health facility based studies, respondents are likely to be medical patients who may be reporting somatic symptoms (e.g. fatigue and anorexia) that might be confounded by the underlying condition that the patients are seeking care. Spitzer et al. [55] recommended that tools with questions about appetite, fatigue, or sleep (e.g., PHQ-9) must be interpreted cautiously, as impairment might reflect the physical effect of pregnancy rather than depressive symptoms.

We found a statistically significant difference in prenatal depressive symptoms prevalence among the three study districts with the lowest prevalence found in Woliso. Worldwide estimates of depressive symptoms vary widely between studies and settings, discrepancies being attributable to real differences between countries but also to the method of assessment [56]. Previous studies in Ethiopia reported a differential prenatal depressive symptoms prevalence by study sites [4–7]. Each of these studies used different tools to screen depressive symptoms.

Adjusting for relevant confounding variables, we found that marital status, geographical location, family size, household food insecurity and anaemia were identified as predictors of prenatal depressive symptoms. The association between marital status and prenatal depressed mood is consistent with a number of studies in low, middle and high income countries where they found higher rates of mental distress in the widowed, separated and divorced women in comparison with married women [57, 58]. However, other general population studies reported no association [59, 60].

The main strength of this study lies in access to community based data to describe prevalence and associated risk factors of depressive symptoms during pregnancy. This study is also based on large sample size and huge response rate; a very thorough description of the population with a big number of questionnaires on different socio-demographic, nutritional and other clinical risk factors. Being a cross-sectional analysis, the usual restrictions inherent to cross-sectional and observational studies apply here; no information about causality. An additional limitation of this study is that we used one month recall on the food-insecurity measure, but a two weeks recall on the measure of depressive symptoms, which raises concerns over the reported associations.

## Conclusions

Prenatal depressive symptomatology is quite common during pregnancy. Socio-demographic factors such as marital status, family size and geographical location are associated with an increased prevalence of prenatal depressive symptoms. Similarly, nutrition related factors such as household food insecurity and anaemia are associated with prenatal depression. While social support from friends, families and spouse during pregnancy are protective, intimate partner violence augments prenatal depression.

The implications of our study for practice are to emphasize the need for targeted screening for intimate partner violence and depressive symptoms during pregnancy and to link cases to health facilities where treatment is available. In this regard we recommend the Ethiopian Ministry of Health to integrate screening of depressive symptoms and intimate partner violence in routine antenatal care services. Policies aimed at reducing household food insecurity, maternal anaemia, intimate partner violence and promoting maternal social support are likely to have a significant public health impact in preventing prenatal depression. Organizing a mental health team, including health extension workers, in antenatal services to screen and treat prenatal depression together with the aforementioned risk factors during pregnancy might prevent or ameliorate prenatal depression.

## Abbreviations

CI: Confidence Interval
HITS: Hurt, Insult, Threaten and Scream
IPV: Intimate Partner Violence
LMIC: Lower-Middle-Income Countries
MSS: Maternal Social support
MDD: Major depressive disorder
MUAC: Mid upper arm circumference
OR: Odds Ratio
PHQ-9: Patient health questionnaire-9
PUFA: Polyunsaturated fatty acids
SPSS: Statistical Package for the Social Sciences
USAID: United States Agency for International Development
